# Zooming into the Matrix: Using Nonlinear Optical Microscopy to Visualize Collagen Remodeling in Asthmatic Airways

**DOI:** 10.1164/rccm.201904-0722ED

**Published:** 2019-08-15

**Authors:** Robert J. Snelgrove, Dhiren F. Patel

**Affiliations:** ^1^ National Heart and Lung Institute Imperial College London London, United Kingdom

The extracellular matrix (ECM) is of fundamental importance for the functional capabilities of the lung because it serves to maintain tissue tensile strength, elasticity, and barrier function. Furthermore, it is increasingly apparent that the ECM not only acts as a scaffold for tissue resident cells but also directly orchestrates essential cellular processes, including morphogenesis, signal transduction, migration, proliferation, and wound repair (1). Accordingly, an aberrant ECM, as is frequently seen in chronic lung diseases, is not only an end-stage pathological manifestation that compromises tissue functionality but also likely dictates the development and progression of the disease. Collagen accumulation around the airways of patients with asthma is a hallmark pathological feature of ECM remodeling that is associated with irreversible changes in airway obstruction (2). However, our understanding of these pathological changes is limited to a macroscopic assessment of the gross quantities of collagen as adjudged by historical histological stains, and this tells us very little about how the structural or biochemical properties of the collagen may be aberrant. This oversimplification likely neglects potentially important differences in the organization of the collagen, which could impact its capacity to modulate cellular behaviors and define tissue architecture and functionality.

In a study presented in this issue of the *Journal*, Mostaço-Guidolin and colleagues (pp. 431–443) used nonlinear optical microscopy (NLOM) to assess the biochemical and structural features of collagen and elastin in nontransplantable donor lungs from individuals with and without asthma (3). The nonlinear interactions that are the basis of the images generated by NLOM occur naturally between light and specific biological molecules owing to their intrinsic natural properties, thus allowing these macromolecules to be imaged without the application of exogenous stains. In their elegant study, the authors comprehensively demonstrate that fibrillar collagen was not only increased in mass within the lamina propria of small and large airways of patients with asthma, but also disorganized and fragmented. These changes were apparent in patients with asthma regardless of age or sex, and were comparable between patients with fatal disease and those with nonfatal disease. Rationalizing these changes, the authors demonstrated that fibroblasts isolated from the airways of patients with asthma were defective in collagen I fiber formation relative to those derived from healthy control subjects. They attributed this failing to a reduced expression of decorin (a proteoglycan that is required for normal spacing of collagen fibrils within fibers) by airway fibroblasts from subjects with asthma. In support of this assertion, the authors demonstrated that packaging of collagen fibrils was indeed disorganized in the airways of patients with asthma.

The study by Mostaço-Guidolin and colleagues represents a step change in the manner in which we should now view the ECM in defining a disease state. It is no longer enough to consider the relative levels of ECM macromolecules—we should also consider the potential implications of more subtle differences in morphology and physiology. However, these studies inevitably raise further questions. At present, the postulated mechanism whereby reduced fibroblast decorin expression drives disorganized airway collagen in patients with asthma remains primarily correlative and needs to be proved experimentally. The authors’ assertion that changes in decorin are causal is supported by observations that decorin knockout mice present with collagen fibril disorganization in the skin and tendons (4). Future studies should seek to manipulate decorin levels in murine models of allergic airways disease and primary patient cells to validate that they impact collagen organization and ultimately asthma pathophysiology. Furthermore, it is important to confirm that decorin expression is indeed reduced in fibroblasts proximal to the disorganized airway collagen of patients with asthma, as well as to gain a fuller understanding of the pathways that define its expression and determine whether they can be therapeutically manipulated.

It is interesting that disorganized collagen is observed in the airways of patients with asthma regardless of sex and age, and whether they have fatal or nonfatal disease, but this ultimately raises questions about the role that changes in collagen organization play in driving disease pathology and severity. Analysis of cadavers, although clearly advantageous for whole-lung analysis of ECM by NLOM, has limitations with regard to the availability of paired patient histories. In the future, it would be of interest to correlate these observed changes in collagen organization with underlying inflammation, lung function, disease severity, and, more broadly, patient endotype. This ultimately raises a more generic question as to the physiological significance of disorganized collagen. The abundance of collagen fibers correlates with the resistance and elasticity of parenchymal tissues (5), and the organized assembly of collagen fibrils is of central importance in defining the tensile strength of the collagen (6). However, there is clearly a need to more fully understand the effects of disorganized collagen on airway mechanics and airway hyperresponsiveness. More broadly, given the capacity of ECM macromolecules to regulate pleiotropic cellular activities, it will be intriguing to determine how structural changes in collagen impact its signaling capacity. A more in-depth understanding of the processes that drive disorganized airway collagen in individuals with asthma, and ensuing implications of these changes in regulating airway mechanics and behavior of proximal cells, could ultimately lead to identification of new therapeutic targets.

**Figure 1.**
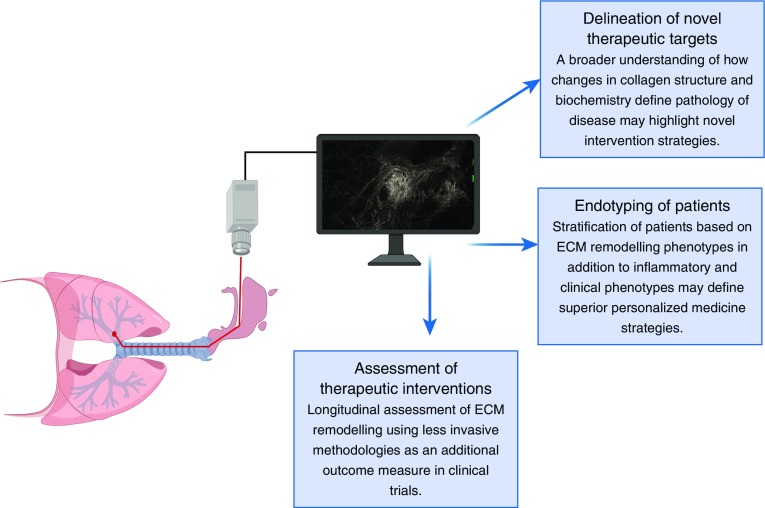
Future clinical applications for nonlinear optical microscopy. The ability to combine nonlinear optical microscopy with bronchoscopy to generate images of extracellular matrix (ECM) macromolecular structure without the use of exogenous stains makes this approach an exciting, less invasive way to longitudinally assess airway remodeling in patients.

The chronic inflammation observed in asthmatic airways was classically believed to be the driver of airway remodeling, but it is now acknowledged that both processes can occur in parallel and independently, and are of comparable importance in defining the clinical course of disease (7). Given the importance of both airway inflammation and remodeling in defining asthma pathogenesis, it is noteworthy that there is significant disparity in the assessment of these dual processes. The vast majority of clinical studies have focused on assessment of the inflammatory rather than airway remodeling profile of patients owing to the challenges of assessing airway remodeling noninvasively, with analysis generally necessitating bronchial biopsies. It is intriguing, therefore, that NLOM has successfully been coupled with endoscopy techniques for the assessment of airways *in vivo* (8), and given that visualization of collagens and elastin by NLOM does not necessitate the addition of exogenous stains, it is clear that this represents a potentially exciting, less invasive technology with clear clinical applications (Figure 1). Ideally, unbiased statistical approaches to define asthma endotypes should incorporate a remodeling phenotype together with routinely assessed clinical and inflammatory parameters, as the contributions and interactions of each factor will ultimately dictate disease outcomes. It is tantalizing to consider that the combination of endoscopy with NLOM technology could lead to a more routine assessment of airway collagen and elastin deposition in individuals with asthma, which could be tracked longitudinally with inflammation and clinical parameters to delineate the evolution of the disease. Therapeutic strategies for asthma are entering an age of personalized medicine (9, 10), and incorporating remodeling phenotypes to stratify patients will be fundamentally important for identifying optimal treatment regimens. Furthermore, assessment of airway remodeling is rarely included as an outcome measure in clinical trials owing to the invasive nature of tissue biopsies, but the use of NLOM could facilitate a longitudinal interrogation of the effects of therapeutic interventions on changes to the ECM and ensuing disease control or remission.
